# Madelung’s disease involving the scrotum: a case report and diagnostic pitfalls with liposarcoma

**DOI:** 10.3389/fonc.2026.1702788

**Published:** 2026-03-05

**Authors:** Shu-Hui Wang, Weiwei Yu

**Affiliations:** Department of Radiology, Weihai Municipal Hosipital, Cheeloo College of Medicine, Weihai, China

**Keywords:** differential diagnosis, liposarcoma, Madelung’s disease, magnetic resonance imaging, multiple symmetric lipomatosis, scrotum

## Abstract

**Background:**

Madelung’s disease is a rare metabolic disorder characterized by diffuse, symmetrical adipose tissue proliferation, strongly associated with chronic alcohol consumption. Scrotal involvement is exceedingly rare and can mimic malignancy.

**Case presentation:**

We report a 59-year-old man with a history of chronic alcohol intake who presented with progressive bilateral scrotal enlargement. Unlike previously reported cases of scrotal Madelung’s disease, which typically demonstrate homogeneous and unencapsulated fat proliferation, MRI in this patient revealed atypical imaging features, including multiple internal fibrous septa and distinct nodular-like foci within fat-containing scrotal masses, raising suspicion of liposarcoma. Complete surgical excision was performed. Histopathology confirmed benign lipomatosis with CDK4(+), MDM2(+), Ki-67 (+,2%), and negative MDM2 amplification on fluorescence *in situ* hybridization (FISH), arguing against well-differentiated liposarcoma. The patient remained recurrence-free at 12-month follow-up.

**Conclusion:**

This case emphasizes the critical role of multimodal medical imaging, rigorous histopathological evaluation, and molecular testing in establishing an accurate diagnosis and guiding appropriate management, while highlighting that Madelung’s disease may occasionally present with atypical imaging features, such as nodules and septa which closely mimic a malignant lesion.

## Introduction

Madelung’s disease, also known as Multiple Symmetric Lipomatosis (MSL), is a rare metabolic disorder characterized by the abnormal proliferation of symmetrical adipose tissue (1). This condition is predominantly associated with chronic alcohol consumption and metabolic disturbances, with a higher incidence in males, particularly those aged 40–60 years. Alcohol may disrupt lipid metabolism, leading to fat accumulation in various tissues (2).

Madelung’s disease is typically classified into two subtypes based on the location and distribution of fat deposits (3). Type 1 is characterized by fat accumulation mainly in the neck, shoulders, and upper arms, forming prominent masses and producing a distinctive ‘buffalo hump’ appearance. Type 2, by contrast, involves more diffuse fat deposition, affecting the trunk and limbs with a more uniform distribution (4). Although rare, involvement of the scrotum has been documented in a limited number of cases. Fat deposition in the scrotum can cause pain, discomfort, and aesthetic concerns, and in severe cases interfere with normal urination or sexual function. Lipomatosis within the scrotum is typically soft, painless masses. If the scrotal fat masses gradually enlarge over time, they may be misdiagnosed as other scrotal lesions, such as hydrocele or testicular tumors, which makes accurate diagnosis through clinical evaluation and imaging essential. Ultrasound is useful for assessing the nature of the scrotal fat masses, typically revealing hypoechoic regions, while CT and MRI provide further insight into the distribution of adipose tissue (5).

**Figure 1:**
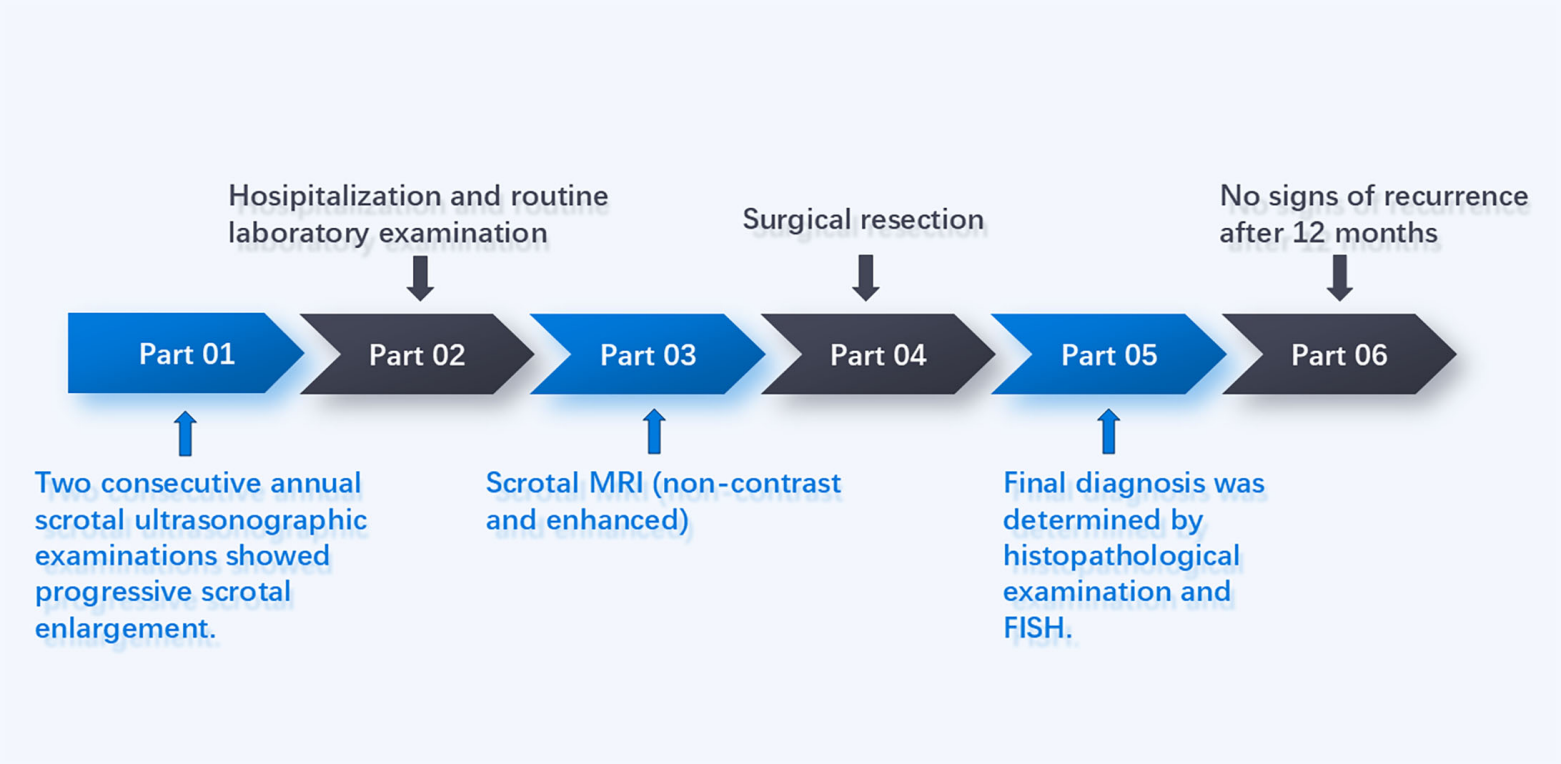
The timeline of diagnosis and treatment for the patient.

Here, we describe a case of Madelung’s disease involving the scrotum that, unlike typical presentations, demonstrated unusual nodular and septal features on MRI. The patient was followed up for 12 months postoperatively, with no signs of recurrence. The timeline of diagnosis and treatment are shown in **Figure 1**.

## Case presentation

A 59-year-old man with a long history of chronic excessive alcohol intake (up to 10 units daily for more than 30 years) was first noted to have a cervical mass in 2015. He was admitted two years later for further evaluation. Physical examination revealed diffuse thickening of the cervical soft tissues with prominent fat accumulation extending to the neck, upper chest, and upper back. Preoperative ultrasonography demonstrated a cervical lesion measuring 1.5cm × 0.6 cm, and surgical excision of the cervical mass was subsequently performed. Histopathological examination revealed mature adipose tissue without cytologic atypia.

In 2023, the patient was re-admitted because of progressive enlargement of a scrotal mass over the preceding four years. Physical examination revealed a massive, bilateral scrotal mass extending to the suprapubic region. The lesion was soft in consistency, poorly demarcated, and moderately mobile, with no overlying skin erythema or tenderness. Due to patient privacy concerns, no clinical photographs were obtained.

The patient underwent two consecutive annual scrotal ultrasonographic examinations. Both testes appeared morphologically and dimensionally normal; however, they were surrounded by hyperechoic masses extending into the perineal region. Interval progression was evident, with a marked increase in the size of the masses during the second examination.

**Figure 2:**
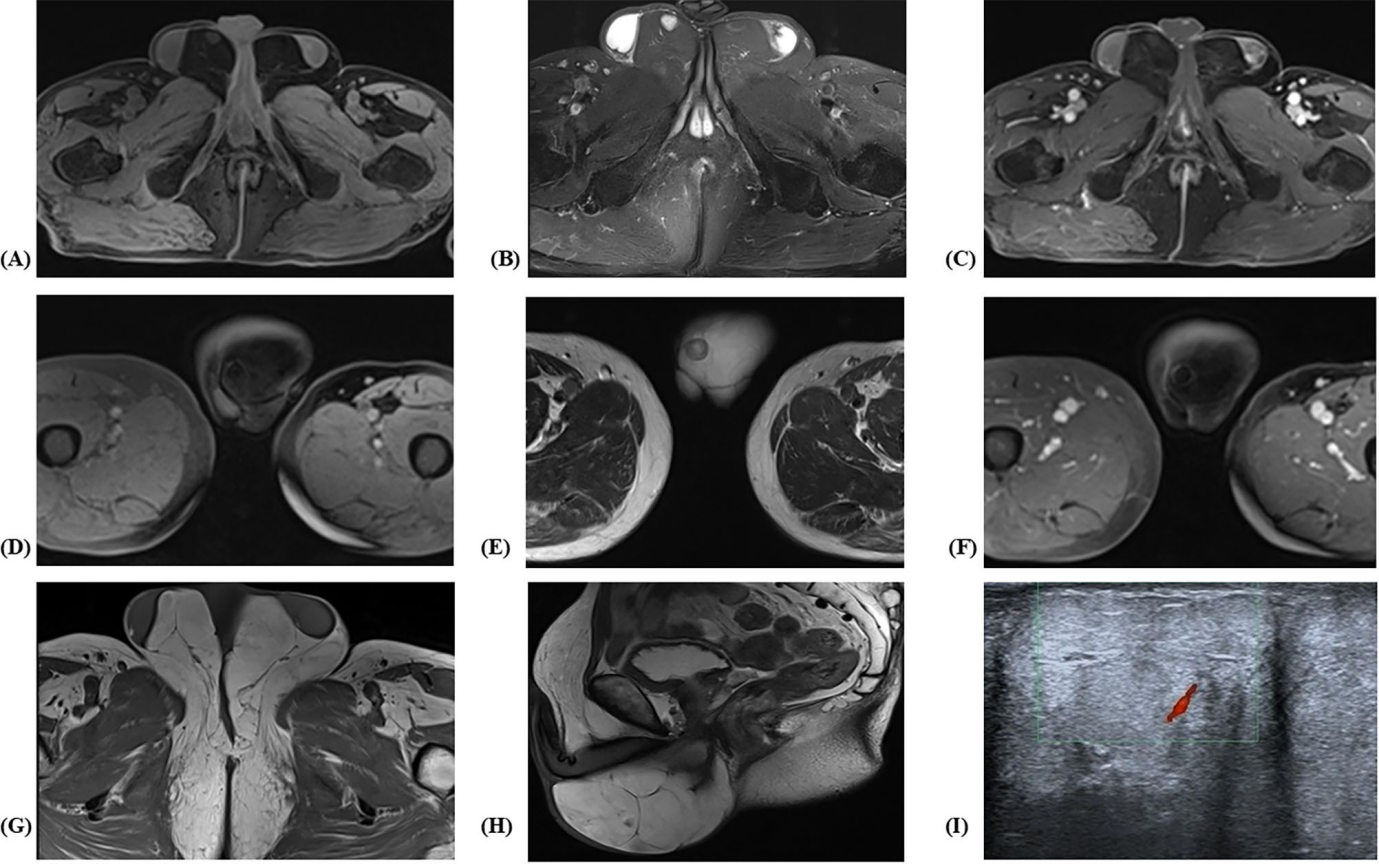
Axial T1WI-FS **(A)**, T2WI-FS **(B)** and T1WI-enhanced **(C)** MR images of the scrotum showed the nodule in right scrotal sac was hyperintense on T2WI and hypointense on T1WI. The small nodule exhibited mild contrast enhancement. Axial T1WI-FS **(D)**, T2WI-FS **(E)** and T1WI-enhanced MR **(F)** images showed the nodule in left scrotal sac was fat signal with ring-like low signal intensity and mild peripheral (ring-like) enhancement. Axial T1WI-FS **(G)** and sagittal T2WI **(H)** demonstrated fat deposition of the perineum and the root of the penis with marked septations. Scrotal ultrasound **(I)** showed two morphologically and dimensionally normal testicles encircled by hyperechoic masses which involved perineum.

The patient underwent two scrotal ultrasonography examinations, both of which suggested a soft tissue lesion. Given the superior soft tissue contrast of MRI for evaluating adipocytic tumors, abdominal and pelvic CT was not performed, and MRI was selected as the primary modality for further assessment. Magnetic resonance imaging (MRI) of the scrotum was subsequently performed using a 3.0-T scanner. The testes were of normal size but displaced laterally by extensive fat-containing masses exhibiting internal low-signal septations. Within these fatty deposits, two nodules were identified, measuring 1.0 cm and 1.2 cm in diameter, respectively. The abnormal fatty proliferation extended continuously into the perineal adipose tissue without clear demarcation and involved the root of the penis (**Figure 2**).

**Figure 3:**
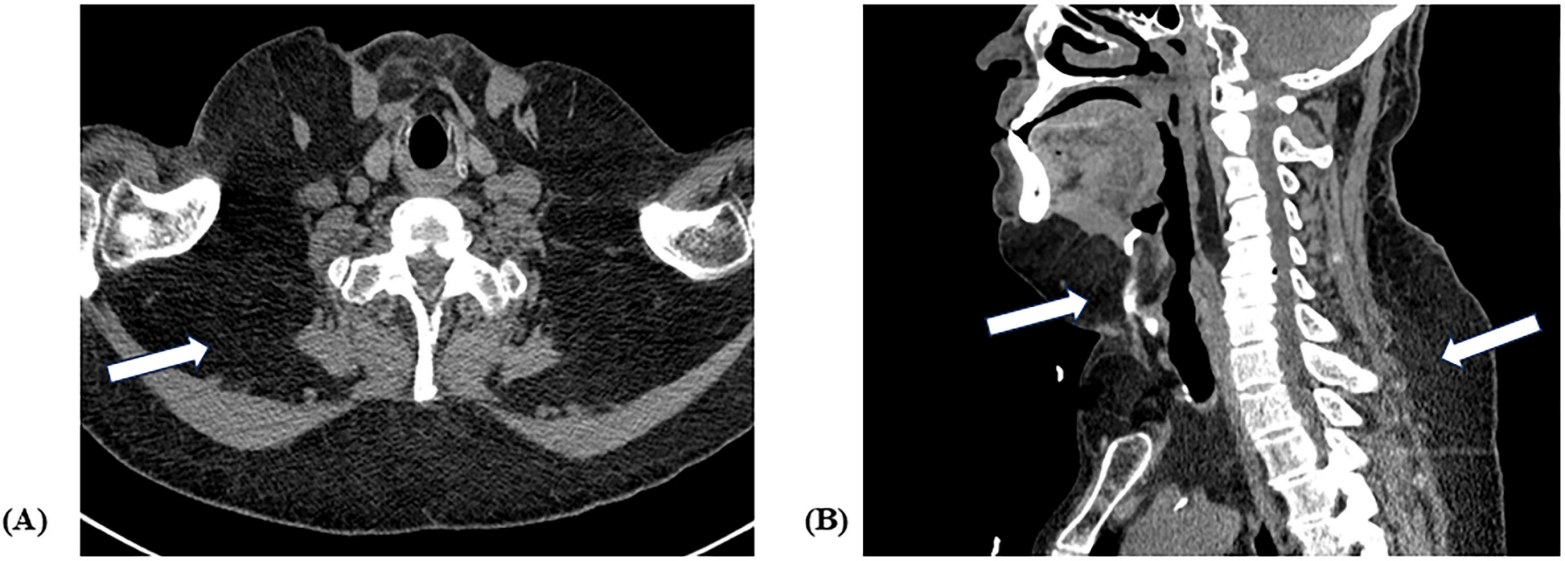
Axial **(A)** and coronal **(B)** CT images of the neck demonstrating diffuse fat accumulation (white arrows) in the anterior and posterior subcutaneous tissues, extending into the thoracic inlet.

Computed tomography (CT) of the neck and chest demonstrated symmetric, nonencapsulated fat deposition with soft-tissue attenuation, predominantly located in the anterior and posterior subcutaneous tissues of the neck and extending into the thoracic inlet. In contrast to the scrotal fat accumulation, the cervical deposits showed neither encapsulation nor internal septations (**Figure 3**).

**Figure 4:**
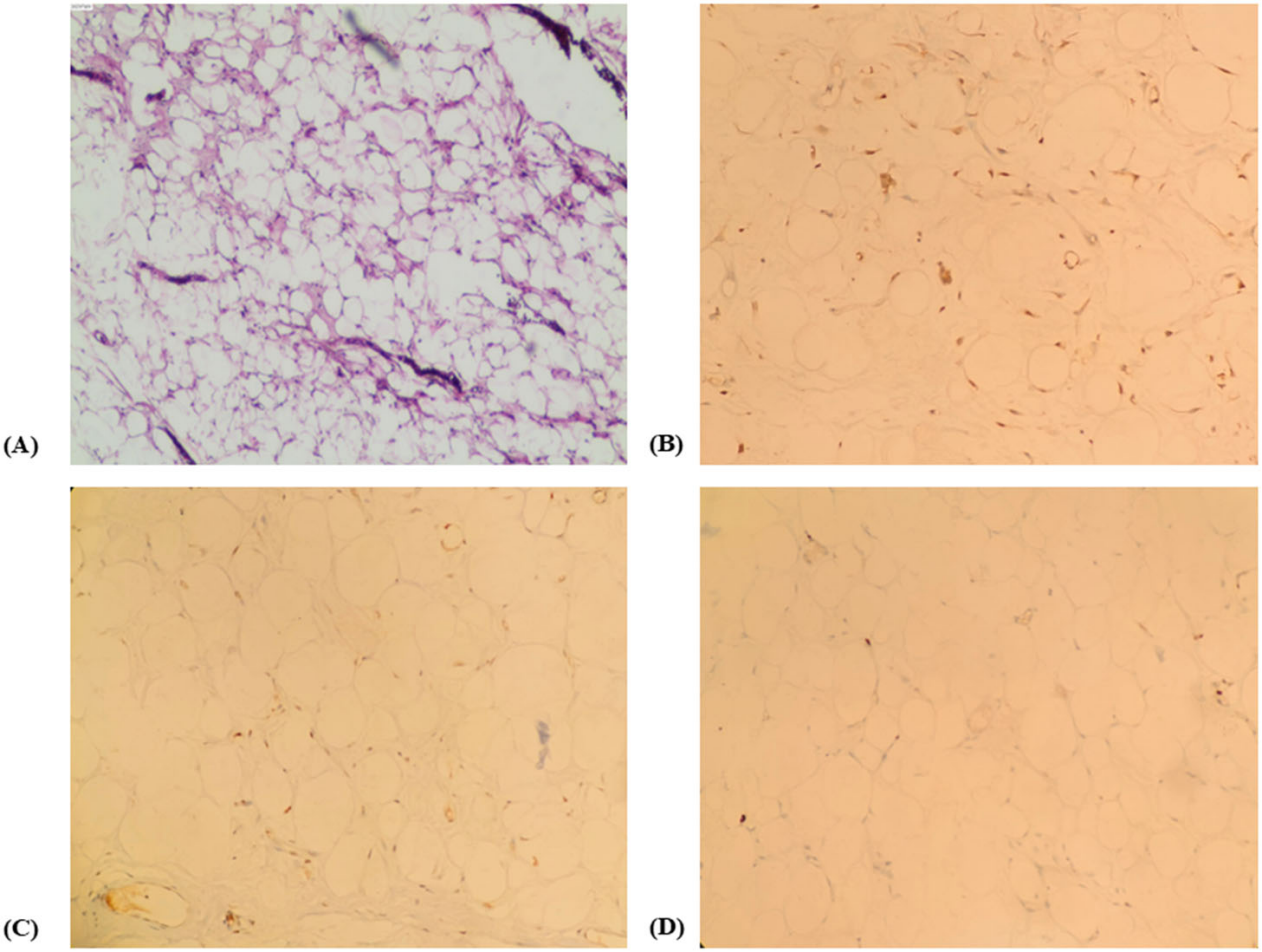
Histopathological and immunohistochemical findings of the scrotal lesion. **(A)** Hematoxylin and eosin (H&E) staining demonstrates proliferation of mature adipose tissue composed of relatively uniform adipocytes, separated by delicate fibrous septa, without significant cytologic atypia, lipoblasts, or increased mitotic activity (original magnification ×200). **(B)** Immunohistochemical staining for MDM2 shows focal and weak nuclear positivity in scattered stromal cells, while the majority of mature adipocytes are negative (original magnification ×200). **(C)** Immunohistochemical staining for CDK4 reveals focal nuclear positivity in a small subset of stromal cells, without diffuse or strong expression (original magnification ×200). **(D)** Ki-67 immunohistochemical staining demonstrates a low labeling index of approximately 2%, indicating minimal proliferative activity and supporting a benign adipocytic process (original magnification ×200).

Histopathological examination of the resected lesions confirmed that no significant cytologic atypia or mitotic activity was observed, consistent with lipomatosis (**Figure 4**). Immunohistochemical staining revealed tumor cells focal positive for CKD4, MDM-2, and S-100, with Ki-67 (+,2%). Fluorescence *in situ* hybridization (FISH) analysis demonstrated no amplification of the MDM2 gene.

Based on the characteristic MRI features of diffuse fat deposition, together with the patient’s history of chronic alcohol consumption, a final diagnosis of multiple symmetric lipomatosis (MSL) was established.

The patient underwent surgical excision of the perineal masses. Postoperatively, recovery was uneventful, with satisfactory cosmetic appearance and preserved functional outcomes. At the 12-month follow-up, no evidence of recurrence was noted.

## Discussion

Scrotal involvement in Madelung’s disease is exceedingly rare, with only a few cases reported in the literature (6), most of which describe homogeneous, non-encapsulated adipose proliferation with imaging characteristics similar to subcutaneous fat. Poggi et al. reported the first detailed description of scrotal involvement using ultrasound and MRI, demonstrating diffuse, unencapsulated fat masses with fine linear strands but without nodular components or thickened septa (5). Subsequent reports, including those by Costa et al. (6) and Nikolić et al. (7), described scrotal Madelung’s disease primarily as a cause of cosmetic deformity or functional impairment such as hidden penis syndrome, with imaging findings remaining largely homogeneous and without features suspicious for malignancy. Surgical outcomes in these cases were generally favorable following lipectomy, with low recurrence rates during follow-up.

The current case showed imaging characteristics that overlap with previously documented presentations, distinct differences are noted. MRI of this patient revealed bilateral scrotal fat proliferation with multiple internal fibrous septa and distinct nodular-like foci—findings rarely described in prior literature. These features were notably absent in the patient’s cervical and upper back lesions, suggesting a region-specific variation in disease progression. The presence of atypical imaging findings in an uncommon location, combined with the rare potential for malignant transformation in Madelung’s disease (as described in isolated case reports (8)), may lead to confusion with liposarcoma.

The scrotum comprises multiple tissue components, including the epididymis, spermatic cord, tunica vaginalis, and a robust fibro-fatty-muscular supporting structure. Although neoplasms arising from these structures are uncommon, they encompass a broad spectrum of histological types. In adults, most scrotal masses are of testicular origin. Sarcomas represent approximately 30% of all scrotal tumors, with liposarcoma (LPS) being the most frequently reported subtype (9). LPS is a malignant mesenchymal neoplasm composed of adipocytic cells with varying degrees of differentiation and cytologic atypia. It accounts for approximately 20% of all soft-tissue sarcomas and typically affects middle-aged to older adults (10). Clinically, LPS may present as a slowly enlarging, painless scrotal mass. Sonographic findings are often nonspecific, typically showing a hyperechoic or heterogeneous mass of uncertain origin. MRI typically demonstrates a heterogeneous lesion with mixed adipose and non-adipose components, raising suspicion of liposarcoma rather than benign lipomatous proliferation. Definitive diagnosis requires histopathologic confirmation, supported by immunohistochemical and cytologic analyses. The identification of fibrous septa, nodular architecture, and non-lipomatous soft-tissue elements on imaging may further aid in distinguishing LPS from benign entities such as lipoma or diffuse lipomatosis.

Immunohistochemical analysis in this case demonstrated positive expression of CDK4 and MDM2, suggesting aberrant adipocytic proliferation. It is noteworthy that Madelung’s disease, characterized by diffuse symmetrical lipomatosis, typically exhibits mature adipose tissue on histology without expression of tumor-associated markers such as CDK4 or MDM2. While these findings are not definitive for malignancy, CDK4(+) and MDM2(+) raise concern for potential neoplastic behavior. The combined use of CDK4, MDM2, and p16 immunostaining has been established as a sensitive and specific adjunct for distinguishing LPS from other lipomatous tumors (11). In particular, FISH for MDM2 gene amplification remains the gold standard for diagnosing well-differentiated liposarcoma (WDLPS) and dedifferentiated liposarcoma (DDLPS), despite its limited specificity (12). In the present case, FISH testing did not reveal MDM2 gene amplification. Ki-67 immunostaining revealed a low labeling index of approximately 2%, consistent with a benign adipocytic lesion. The low Ki-67 index, together with negative MDM2 amplification, supports a diagnosis of benign lipomatosis rather than a malignant adipocytic neoplasm. However, discordance between MDM2/CDK4 immunohistochemistry and MDM2 fluorescence *in situ* hybridization has been increasingly recognized in the evaluation of adipocytic tumors. Recent studies have demonstrated that MDM2 and CDK4 immunostaining show only moderate sensitivity and specificity when compared with MDM2 FISH as the reference standard. Nomura et al. reported sensitivities of 55.6% for MDM2 and 40.0% for CDK4 immunostaining, with specificities of 87.0% and 84.6%, respectively, indicating a non-negligible false-positive rate in benign lipomatous lesions (13). In addition, Machado et al. emphasized that immunohistochemical expression of MDM2 and/or CDK4 should be interpreted with caution, as isolated or weak positivity may be observed in a subset of conventional lipomas without underlying gene amplification (14). These findings underscore that immunohistochemistry alone cannot reliably distinguish benign lipomatosis from ALT/WDLPS and that molecular confirmation by FISH remains essential in cases with atypical clinical or radiologic features. In the present case, the absence of MDM2 gene amplification by FISH, together with a low Ki-67 proliferation index, argues against a diagnosis of liposarcoma despite positive CDK4 and MDM2 immunoreactivity. The observed discordance likely reflects the limited specificity of immunohistochemical markers rather than true neoplastic transformation, reinforcing the importance of integrating histology, immunophenotype, molecular testing, and imaging findings in the diagnostic assessment.

Postoperative histopathological analysis did not identify the small nodules previously observed in preoperative MRI. Several potential explanations may account for this radiologic-pathologic discrepancy. First, the nodules on MRI may have represented internal fibrous septa, vascular structures, or other microscopic components within the adipose tissue. These subtle architectural features often produce nodular or reticulated signal patterns, particularly on T1WI and T2WI, but are not typically highlighted or specifically identified during routine histopathologic evaluation, especially when they lack overt pathological significance. Second, the MRI-detected nodules could have corresponded to minute benign lesions, such as small lipomas or fibrous nodules, that were either not included in the resected specimen or not captured in the sampled histologic sections. Histopathologic findings are inherently dependent on both the extent of surgical resection and the planes of tissue sectioning (15). If the resected tissue did not precisely encompass the MRI-identified nodular regions, or if the sectioning planes did not traverse the relevant areas, the corresponding lesions may have been inadvertently missed during pathological examination. This type of mismatch between imaging and pathology is not uncommon in cases involving large or diffuse resections, where spatial correspondence between radiologic and histologic findings can be challenging to establish.

Although Madelung’s disease is generally considered a benign disorder characterized by adipose tissue accumulation, several studies have documented its potential for malignant transformation. Tizian et al. reported a case of cervical Madelung’s disease that progressed to myxoid liposarcoma (16). Later, Borriello et al. described a breast cancer patient who developed a well-differentiated liposarcoma in the region affected by Madelung’s disease, raising concern about the coexistence of dual malignancies (8). More recently, Lungu et al. (3)reported malignant transformation with hepatic metastases in a patient with Madelung’s disease, who ultimately succumbed to tumor progression. Imaging plays a pivotal role in differentiating benign from malignant lipomatous lesions. MRI features, in particular, are of significant diagnostic value in distinguishing lipoma from atypical lipomatous tumor/well-differentiated liposarcoma (ALT/WDL). In a retrospective analysis of 79 lipomatous tumors, Moran et al. found that homogeneous fat signal, absence of septa, and a lesion diameter of <8 cm were highly suggestive of lipoma. Conversely, the presence of septa≥2 mm in thickness, multiple non-adipose nodules, and a maximum tumor diameter of ≥12.8 cm strongly indicated ALT/WDL (17).

The patient had a history of chronic alcohol consumption and exhibited prominent fat accumulation in the neck and thoracic inlet, consistent with a diagnosis of Madelung’s disease. The previously resected cervical mass was histologically composed of mature adipose tissue. In retrospect, considering the patient’s chronic alcohol abuse and subsequent disease course, this lesion may represent fat accumulation associated with Madelung’s disease rather than a sporadic lipoma. The patient’s distribution of adipose proliferation involved typical anatomical regions for Madelung’s disease, including the neck, upper trunk, and proximal perineal region, consistent with the known pattern of diffuse, symmetric fat deposition. The temporal progression—from cervical involvement to subsequent scrotal and perineal enlargement over several years—further supports a systemic rather than localized process. Compared with previous literature, the present case demonstrated atypical features, including progressive bilateral scrotal involvement and the presence of suspicious nodules and multiple internal septa within the lipomatous masses on MRI. Differentiating between benign lipomatosis and liposarcoma—two entities that share a similar adipocytic lineage—is of particular clinical importance, especially when lesions exhibit atypical growth patterns or imaging features suggestive of solid components.

Although liposuction has been described as an effective treatment option for less extensive and homogeneous fat accumulation in Madelung’s disease, particularly in cosmetically driven cases, it does not allow adequate histopathological assessment and may be insufficient when malignancy cannot be confidently excluded (7). Given the rare localization of Madelung’s disease involving the bilateral scrotal regions, together with suspicious MRI findings in this patient, the possibility of an underlying or coexisting malignancy had to be considered in the differential diagnosis. Therefore, complete surgical excision was selected to achieve definitive diagnosis and ensure oncologic safety. Regardless of malignant potential, the persistent enlargement of the lesions in this patient warranted surgical intervention. Surgery remains the preferred treatment option in such cases, with the goals of improving cosmetic appearance, relieving potential compression of critical structures, and preventing functional impairment, such as sexual dysfunction or urinary obstruction (18).

## Conclusion

Madelung’s disease is typically characterized by symmetric, non-encapsulated, diffuse adipose tissue deposition with indistinct margins that may extend into adjacent structures. In this case, scrotal involvement was accompanied by atypical imaging features, including fibrous septa and small nodules, closely mimicking liposarcoma. This highlights the critical role of multimodal medical imaging, rigorous histopathological evaluation, and molecular testing in establishing an accurate diagnosis and guiding appropriate management.

## Data Availability

The raw data supporting the conclusions of this article will be made available by the authors, without undue reservation.
